# Prevalence of oral human papillomavirus infection among Indian HIV-positive men who have sex with men: a cross-sectional study

**DOI:** 10.1186/s12879-021-06301-6

**Published:** 2021-07-12

**Authors:** Alexandra L. Hernandez, Rajiv Karthik, Murugesan Sivasubramanian, Anantharam Raghavendran, Shelly Lensing, Jeannette Y. Lee, Priya Abraham, Dilip Mathai, Joel M. Palefsky

**Affiliations:** 1 Division of Infectious Diseases, Department of Medicine, University of California, Box 0654, 513 Parnassus Ave, Room S420, San Francisco, CA 94143 USA; 2 Public Health Program, College of Education and Health Sciences, Touro University, Vallejo, CA USA; 3 Department of Infectious Diseases, Christian Medical College, Vellore, India; 4 The Humsafar Trust, Mumbai, India; 5 Department of Clinical Virology, Christian Medical College, Vellore, India; 6 Department of Biostatistics, University of Arkansas for Medical Sciences, Little Rock, AR USA; 7 Apollo Institute of Medical Sciences and Research, Hyderabad, India

**Keywords:** Human papillomavirus, HPV, HIV/AIDS, Oral cancer, MSM

## Abstract

**Background:**

Oral human papillomavirus (HPV) infection has been causally linked to a subset of oropharyngeal cancers in Western populations, and both oropharyngeal cancer and oral HPV infection are increased among HIV-positive individuals. India has high incidences of oral and oropharyngeal cancers, and Indian HIV-positive men who have sex with men (MSM) may be at increased risk of developing oropharyngeal cancers. However, there is little information available on the prevalence of oral HPV in this population.

**Methods:**

We tested 302 HIV-positive Indian MSM for oral HPV infection using L1 HPV DNA PCR with probes specific for 29 types and a mixture of 10 additional types. CD4+ level and plasma HIV viral load (VL) were measured. Participants completed an interviewer-administered questionnaire including a sexual history.

**Results:**

The prevalence of oral HPV was 23.7% (95% CI: 19–29%) and 2.4% of participants had oncogenic HPV types. No participants had oral HPV type 16 (HPV-16) and the prevalence of other anogenital HPV types was low. Participants with higher CD4+ levels had reduced odds of having any oral HPV infection (OR: 3.1 [1.4–6.9]) in multivariable analyses.

**Conclusions:**

This is the first report of oral HPV among Indian HIV-positive MSM. Our results show a high prevalence of oral HPV infection consistent with studies from Western populations. However, oncogenic anogenital HPV types were relatively uncommon in our study population. It is unknown what the impact of this distribution of oral HPV will be on oropharyngeal cancers. HIV-positive MSM in India should be monitored closely for oral and oropharyngeal pre-cancer and cancer.

**Supplementary Information:**

The online version contains supplementary material available at 10.1186/s12879-021-06301-6.

## Introduction

The incidence of oral squamous cell carcinoma (OSCC) is higher in India than in western countries. In India, there are an estimated 12.6–20.0 per 100,000 new cases of OSCC each year [1, 2]. OSCCs comprise 40–50% of all malignancies diagnosed in India and they are the most common cancer among Indian men [3]. This is in contrast to the global prevalence where oral cancers comprise only 2–4% of all cancers, and to the United States where cancers of the oral cavity and pharynx account for only 3% of all new cancer cases [4–9]. The difference in occurrence has largely been attributed to the high prevalence of established risk factors for OSCC, primarily the use of tobacco products and heavy alcohol consumption [10]. India has an additional lifestyle behavior that has been linked to oral cancers, i.e., the practice of chewing ‘betel quid’ (a combination of betel leaf, smokeless tobacco, areca nut, and lime paste). In addition to the established carcinogenicity of the smokeless tobacco in betel quid, areca nut (alone and in combination with the lime paste) is thought to be an oral carcinogen [11–13].

There is established epidemiological and molecular evidence that human papillomavirus (HPV) is causally associated with a subset of OSCCs, oropharyngeal cancer, and particularly with cancers of the palatine and lingual tonsils [14–19]. The prevalence of HPV DNA detected in OSCC varies broadly by DNA detection techniques, population, type of cancer specimen, and anatomical location of the cancer. Worldwide, the prevalence of HPV DNA reported for OSCC is between 11 and 48% [14, 20, 21]. However, HPV is consistently and most frequently detected in cancers of the lingual and palatine tonsils of the oropharynx (12–63%) in studies conducted in the West [17, 22, 23]. HPV-16 is the most common HPV type and accounts for almost 90% of HPV associated OSCCs [16, 24, 25]. Other HPV types found in OSCC include HPV-18, HPV-33, and HPV-35 [16, 24, 25]. Studies conducted in India on HPV in OSCCs show the same variability in estimates as do Western studies (25–100%) [13, 26–33]. Currently, there are few data available on oral HPV infection in individuals without OSCC in India.

HIV infection is associated with increased risk of both OSCC and oral HPV infection in western countries. It is well established that HIV-positive individuals have an elevated risk for HPV-associated cancers including cervical and anal cancers [34]. Similarly, HIV-positive individuals have two to four times the risk of developing OSCC and two to six times the risk of developing oropharyngeal cancer compared with HIV-negative individuals [23]. The incidence of oropharyngeal cancer has not decreased and may be increasing since the advent of antiretroviral medications [35, 36], a relationship that has also been noted with cervical and anal cancers [37]. Also, in western countries, oral HPV infection (as well as anogenital HPV infection) is more common among individuals with HIV infection [38, 39]. HIV-positive individuals have two to three times the risk of oral HPV infection compared with HIV-negative individuals [40] and the prevalence of oral HPV infection has been shown to increase with decreasing CD4+ levels [41]. A recent meta-analysis reported the pooled prevalence of any HPV infection among HIV-positive MSM was 28.9 and 4.7% for HPV-16 [42].

India has approximately 2.14 million individuals living with HIV/AIDS, with Indian MSM having some of the highest prevalence estimates of HIV infection (6–68%) [3, 43, 44]. This confluence of risk factors, including the high background incidence of OSCCs; HIV and HPV infections; and the use of tobacco, alcohol, and betel quid, potentially puts Indian MSM at a uniquely high risk of OSCC. We have also previously reported that this population has a very high prevalence of anal HPV infection [45] and a high prevalence of penile HPV infection [46]. However, there are no studies yet reported on the occurrence of oral HPV infection among Indian HIV-positive men, including MSM. Given that HPV infection is potentially preventable though behavioral modification or vaccination [18, 47], it can be reasoned that a portion of OSCCs may also be preventable. Determining the prevalence of oral HPV infection is an important first step in developing prevention interventions for this high-risk group in India, as well as prevention interventions for HIV-positive individuals worldwide. Therefore, we conducted a cross-sectional study in two cities in India to determine the prevalence and risk factors for oral HPV infection among Indian HIV-positive MSM.

## Materials and methods

HIV-positive MSM were recruited from two study sites, Christian Medical College (CMC), Vellore, Tamil Nadu (a large research and teaching institution) and Humsafar Trust (HT), Mumbai (a male sexual health non-governmental organization [NGO]). Men were recruited through outreach workers, local HIV/AIDS support groups, and were also referred from other NGOs. Enrollment occurred from September 2009 to August 2010. Men were eligible for the study if they had had sexual contact with another male in the preceding 6 months, were HIV-positive, and were at least 18 years of age. Participants were asked to rinse and gargle with 10 ml of Scope™ for 30 seconds and expectorate into a 50 ml wide-mouth sterile tube to collect oral cells for HPV testing as described previously [48]. Participants completed a questionnaire in the local language administered by a male interviewer. The English language version of the questionnaire is included as Supplemental File 1. The participants had a clinical examination, including collection of anal and penile specimens for HPV testing. Blood was collected for a CD4+ lymphocyte count measured by standardized two- or three-color fluorescence methods. Plasma HIV viral load (HIV VL) was measured using the Amplicor HIV Monitor test, Version 1.5 (Roche, USA). All procedures were performed after obtaining written informed consent from participants. The study was approved by the Committee on Human Research of UCSF, and the Institutional Review Boards of both CMC and HT.

Testing for oral, penile and anal HPV DNA was performed as described previously using the polymerase chain reaction (PCR) with L1 consensus primers and probes specific for 29 individual HPV types and a mixture of 10 additional types (using a combined probe) for a total of 39 HPV types [49]. Beta-globin-negative samples (indicating insufficient good quality DNA) were excluded from analysis. We defined infection with an oncogenic HPV type as a positive test for at least one of the following types: 16, 18, 31, 33, 35, 39, 45, 51, 52, 56, 58, 59, 68, 73, and 82 [50]. Results for penile and anal HPV infections were published separately [51, 52].

### Assessment of potential risk factors

Demographic factors, medical history, use of antiretroviral therapy (ART) and history of sexual behavior were collected. We asked the participants if they had smoked more than 100 cigarettes in their lifetime (yes/no). They were asked if they ‘regularly’ chewed smokeless tobacco, and if so how much they used (‘regularly’ was not defined for participants, but was translated into Hindi and Tamil, with the back translation of roughly “regularly” or “often”). We also asked men if they had ever consumed alcoholic beverages. We asked about sexual behaviors over the participant’s lifetime. Men were queried about multiple types of sexual behaviors, including sex with men and women, oral sex with women (participant’s mouth in contact with woman’s vulva/vagina), oral sex with men (participant’s mouth in contact with man’s penis), oral-anal contact (“rimming”, participant’s mouth in contact with partner’s anus), “insertive” anal intercourse (participant inserts his penis into partner’s anus), “receptive” anal intercourse (participant receives his partner’s penis into his anus), and vaginal sex.

### Statistical analysis

Prevalence was calculated as the total number of participants with a positive consensus probe for oral HPV infection divided by the total number of participants positive for beta-globin (multiplied by 100). Exact binomial 95% confidence intervals (CIs) were calculated for each prevalence estimate. We chose to examine bivariable association with factors that had been shown in previous reported studies to be associated with oral cancer, oral HPV infection, or other HPV infections or HPV-associated cancers. We assessed bivariable associations with any oral HPV infection and socio-demographic, clinical and lifestyle characteristics using the chi-square test for categorical variables and analysis of variance (ANOVA) or ranked ANOVAs for continuous variables, as appropriate.

Odds ratios (ORs) were derived from unadjusted logistic regression models. We evaluated confounding between select risk factors that showed significance in bivariable analyses and oral HPV infection by adding covariates to our unadjusted model to construct four additional adjusted models. The first adjusted model contained the select risk factor and the CD4+ level. The second adjusted model contained the risk factor plus the demographic factors age, marital status, and income. The third adjusted model included the risk factor plus the lifetime number of vaginal sex partners. The last adjusted model included the risk factor plus the lifetime number of male partners with whom the participant was the receptive partner during anal intercourse. Potential confounding was considered to be present if the OR on the select risk factor changed by 10% or greater with the addition of the covariable(s) into the model.

Lastly, we constructed a final multivariable model that included variables with a *p*-value of < 0.10 in bivariable analyses, plus any variables which demonstrated confounding. Variables were considered significantly associated with oral HPV infection if the p was < 0.05 (no adjustment was made for multiple comparisons). All analyses were conducted separately by two analysists (ALH and SL) in SAS 9.4 (SAS North Carolina).

## Results

We enrolled 302 HIV-positive Indian MSM and of these, 7 (2%) had beta-globin-negative samples and were excluded from further analysis. Of the 295 remaining participants, the median age was 34 years, the median monthly income was 3000 rupees (approximately $50), and most (65%) had 1–10 years of education. Participants from our two clinical sites were different demographically. Men from Humsafar Trust were younger (38 vs. 32 years), a greater proportion had completed more than 10 years of school (27% vs 9%), they had a higher median income (6000 INR vs. 2000 INR), and more reported their religion as being “Muslim” (24% vs. 3%) (all comparisons *p* ≤ 0.05).

Almost half of our participants reported being married to a woman (47%). Only 30% of men reported that they have ever smoked 100 or more cigarettes, and only 31% reporting chewing tobacco ‘regularly’. More than half of our participants reported consuming alcoholic beverages in their lifetime, but 46% drank less than 1 day per week.

The median CD4+ level of the study population was 424 cells/uL (interquartile range [IQR]: 273–581 cells/uL) and the median HIV VL was 8307 IU/mL (IQR: ≤400–79,400 IU/mL) 36% had undetectable levels. Forty-eight percent of participants were taking antiretroviral therapy (ART) and of those 69% of participants had been taking ART for more than a year. Men from the two different sites did not differ significantly in their lifestyle behaviors or HIV disease status.

### Prevalence of oral HPV infection

The prevalence of oral HPV infection among our participants was 23.7% (95% CI: 19–29%) (Table 1). Three percent of participants had oral infection with oncogenic HPV types. Of the 70 men with positive consensus probes for oral HPV infection, only 17 had positive results on HPV type-specific tests included in our testing. Of the 17 who had HPV type-specific results, 14 (82%) had more than one type of HPV detectable. Oral HPV 16 infection was not detected in any of our participants. The prevalence of oral HPV infection did not differ by study location.

**Table 1:** Type-specific prevalence of oral HPV infection among HIV-positive Indian MSM

HPV Type	Number positive	Proportion of Positives^a^ (% of 70)	Prevalence (% of 295)
Any Type	70	(100)	(24)
6	1	(1.4)	(0.3)
11	1	(1.4)	(0.3)
**18**	**1**	**(1.4)**	**(0.3)**
30	1	(1.4)	(0.3)
32/42	1	(1.4)	(0.3)
**33**	**2**	**(2.9)**	**(0.7)**
**35**	**2**	**(2.9)**	**(0.7)**
**51**	**1**	**(1.4)**	**(0.3)**
57/27	1	(1.4)	(0.3)
**68**	**1**	**(1.4)**	**(0.3)**
72	2	(2.9)	(0.7)
84	1	(1.4)	(0.3)
85	1	(1.4)	(0.3)
90/106	4	(5.7)	(1.4)
**Total with Oncogenic HPV Types**	7	(10)	(2.4)

^a^ Proportion of samples that were positive for specified HPV type out of those who were positive for the consensus probe. HPV types in bold are considered oncogenic [50]

### Unadjusted associations between demographic factors, lifestyle factors, HIV-related factors and oral HPV infection

There was no significant association between any of the demographic factors examined and oral HPV infection (Table 2). Of the lifestyle factors investigated, two factors commonly associated with OSCC, cigarette smoking and alcohol consumption, were not associated with oral HPV infection in this population. However, chewing tobacco ‘regularly’ was associated with lower prevalence of oral HPV infection among HIV-positive MSM (14%) compared with men who did not chew tobacco (28%) (*p* = 0.01, OR 0.4 [95% CI 0.2–0.8]). However, among the 93 men who did chew tobacco regularly, when we examined the frequency that they chewed tobacco by week, the prevalence of oral HPV infection increased with increasing frequency of weekly chewing tobacco use.

**Table 2:** Socio-demographic factors, medical history and oral HPV infection among Indian HIV-positive MSM (*N* = 295)

	No Oral HPV infection		Oral HPV Infection			
Characteristic	N	(%)	N	(%)	***P***-value^d^	OR (95% CI)^d^
**Total N**	**225**	**(76)**	**70**	**(24)**		
Study Site					0.2604	
CMC, Vellore	117	(79)	31	(21)		1.0
HT, Mumbai	108	(73)	39	(27)		1.4 (0.8–2.3)
Age Categories^a^					0.5064	
18–25	33	(79)	9	(21)		1.0
26–35	92	(79)	24	(21)		1.0 (0.4–2.3)
35+	93	(73)	34	(27)		1.3 (0.6–3.1)
Highest number of years of school completed					0.7635	
None	40	(80)	10	(20)		1.0
1–10	145	(76)	46	(24)		1.3 (0.6–2.7)
10+	40	(74)	14	(26)		1.4 (0.6–3.5)
Monthly income (mean ± SD)	4268	(±4128)	5621	(±6320)	0.0970	1.0 (1.0–1.0)
Religion					0.4865	
Hindu	175	(75)	58	(25)		1.0
Muslim	31	(77)	9	(23)		0.9 (0.4–1.9)
Other	19	(86)	3	(14)		0.5 (0.1–1.7)
Marital Status			.		0.9519	
Unmarried	118	(76)	37	(24)		1.0
Married	107	(76)	33	(24)		1.0 (0.6–1.7)
Smoked more than 100 lifetime cigarettes			.		0.5269	
No	160	(77)	47	(23)		1.0
Yes	65	(74)	23	(26)		1.2 (0.7–2.1)
Mean years smoked	4	(±8)	4	(±7)	0.8550	1.0 (1.0–1.0)
Chews tobacco ‘regularly’^b^			.		0.0077	
No	145	(72)	57	(28)		1.0
Yes	80	(86)	13	(14)		0.4 (0.2–0.8)
If Yes: How often chew tobacco on average per week					0.0709	
< 2 days/week	22	(100)	0	(0)		NE
3–5 days/week	9	(90)	1	(10)		1.0
6–7 days/week	49	(80)	12	(20)		7.6 (0.9–61.3)^e^
Consumes alcohol					0.2531	
No	95	(73)	35	(27)		1.0
Yes	130	(79)	35	(21)		0.7 (0.4–1.3)
CD4 (cells/uL)^c^			.		0.0106	
< 200	21	(57)	16	(43)		3.1 (1.4–6.9)
200–500	113	(78)	31	(22)		1.1 (0.6–2.0)
500+	89	(80)	22	(20)		1.0
HIV viral load^c^			.		0.4570	
Undetectable	77	(73)	28	(27)		1.0
> 400–37,400	72	(81)	17	(19)		0.6 (0.3–1.3)
> 37,400	75	(76)	24	(24)		0.9 (0.5–1.7)
Antiretroviral therapy use					0.2227	
No	128	(79)	34	(21)		1.0
Yes	97	(73)	36	(27)		1.4 (0.8–2.4)

^a^ Indicates 1–5% of participant data were missing

^b^ ‘regularly’ was not defined to participants but was translated into Hindi and Tamil, with the back translation of “regularly” or “often”

^c^ Indicates 1–2 individual participant data were missing

^d^ *p*-value for categorical variable from chi-square, and from ANOVA or ranked ANOVA for normally and non-normally distributed continuous variables. Odds ratio (OR) and 95% confidence interval (CI) from unadjusted logistic regression model

^e^ comparison between participants who reported 2–5 days a week and 6–7 days a week. Participants with < 2 days a week were omitted from this calculation

Men with lower CD4+ levels had a higher prevalence of oral HPV infection when compared with men with higher CD4+ levels (500+ cells/uL). Men with < 200 cells/uL had a prevalence of 43% and men with 500+ cells/uL had a prevalence of 20% (*p* = 0.01). HIV VL and ART use were not associated with oral HPV infection.

### Sexual behavior and oral HPV infection

Several oral sexual behaviors were associated with a reduction in prevalence of oral HPV infection among our participants (Table 3). Men who reported having performed oral sex on a woman in their lifetime had a lower prevalence of oral HPV compared with men who reported never performing oral sex on a woman (17% vs. 32%, *p* = 0.02). However, among those reporting that they had performed oral sex on a woman, the prevalence of oral HPV increased with increasing number of partners on whom the participant had performed oral sex. Engaging in oral-anal contact (rimming), showed a similar association with a lower prevalence among those who engaged in this behavior. Ever performing genital oral sex on a male partner was not associated with oral HPV infection. Behaviors that measured non-oral sexual contact with other partners were not associated with oral HPV infection including vaginal sex, total number of male partners, insertive anal intercourse with men, or receptive anal intercourse with men.

**Table 3:** Sexual history and oral HPV infection among Indian HIV-positive MSM

	No oral HPV infection		Oral HPV Infection			
Characteristic	N	(%)	N	(%)	***P***-value^b^	OR (95% CI)^b^
**Total N**	**225**	**(76)**	**70**	**(24)**		
Number vaginal sex partners, lifetime^a^					0.3431	
0	89	(79)	24	(21)		1.0
1–4	65	(70)	28	(30)		1.6 (0.8–3.0)
5–39	33	(82)	7	(18)		0.8 (0.3–2.0)
40+	37	(77)	11	(23)		1.1 (0.5–2.5)
Performed oral sex on women, lifetime					0.0466	
No sexual contact with women	89	(79)	24	(21)		1.0
Sexual contact with women but no oral sex	65	(68)	31	(32)		1.8 (0.9–3.3)
Sexual contact with women and had oral sex	71	(83)	15	(17)		0.8 (0.4–1.1)
If had sexual contact with women:					0.0217	
No oral sex with women	65	(68)	31	(32)		1.0
Oral sex with women	71	(83)	15	(17)		0.4 (0.2–0.9)
If had oral sex: Number of women partners performed oral sex, lifetime					0.2845	
1–4	29	(91)	3	(9)		1.0
5–39	17	(81)	4	(19)		2.3 (0.5–11.4)
40+	25	(76)	8	(24)		3.1 (0.7–12.9)
Number male partners with whom participant was *receptive* partner during anal intercourse, lifetime^a^					0.9665	
1–1000	71	(76)	22	(24)		1.0
1000+	153	(76)	48	(24)		1.0 (0.6–1.8)
Number male partners with whom participant was *insertive* partner during anal intercourse, lifetime					0.0678	
1–100	74	(80)	18	(20)		1.0
101–1000	45	(64)	25	(36)		2.3 (1.1–4.6)
1000+	16	(70)	7	(30)		1.8 (0.6–5.0)
Ever engaged in oral-anal contact, lifetime (either giving or receiving)					0.0481	
No	95	(71)	39	(29)		1.0
Yes	130	(81)	31	(19)		0.6 (0.3–1.0)
Number oral-anal male partners where participant performed *oral* contact, lifetime					0.0118	
0	129	(70)	54	(30)		1.0
1–10	28	(85)	5	(15)		0.4 (0.2–1.2)
11+	68	(86)	11	(14)		0.4 (0.2–0.8)
Perform genital oral sex on male, lifetime	.		.		0.9745	
No	35	(76)	11	(24)		1.0
Yes	190	(76)	59	(24)		1.0 (0.5–2.1)
If Yes: Number genital oral sex male partners where participant performed oral sex, lifetime					0.8506	
1–100	52	(79)	14	(21)		1.0
101–1000	72	(75)	24	(25)		1.2 (0.6–2.6)
1000+	66	(76)	21	(24)		1.2 (0.5–2.5)

^a^ Indicates 1–2 individual participant data were missing

^b^ *p*-value for categorical variable from chi-square, and from ANOVA or ranked ANOVA for normally and non-normally distributed continuous variables. OR, odds ratio and 95% CI, confidence interval from unadjusted logistic regression model. NE, not estimable

^c^ Indicates 1–5% of participant data were missing

### Evaluation of confounding of select risk factors

We evaluated potential confounding between three risk factors and oral HPV infection (Table 4). We found no evidence of confounding with any of the variables examined in the association between performing oral sex on a woman and engaging in oral-anal contact and oral HPV infection. The addition of vaginal sex to the model with chewing tobacco regularly strengthened the association. None of the potential confounding factors examined in this analysis nullified the associations between the chewing tobacco and oral HPV infection or performing oral sex on a woman and oral HPV infection, and all of the 95% CIs continued to exclude the null value of 1.0.

**Table 4:** Potential confounding of the relationship between select risk factors and oral HPV infection among HIV-positive Indian MSM

	Unadjusted	Adjusted for CD4+ level	Adjusted for age, income, and marital status	Adjusted for lifetime vaginal sex	Adjusted for receptive anal sex
Risk factor	OR (95% CI)	OR (95% CI)	OR (95% CI)	OR (95% CI)	OR (95% CI)
Chews tobacco regularly	0.4 (0.2–0.8)	0.4 (0.2–0.8)	0.4 (0.2–0.7)	**0.3 (0.2–0.8)**	0.4 (0.2–0.8)
Performed oral sex on women, lifetime	0.4 (0.2–0.9)	0.4 (0.2–0.9)	0.4 (0.2–0.8)	0.4 (0.2–0.9)	0.4 (0.2–0.9)
Ever engaged in oral-anal contact, lifetime	0.6 (0.3–1.0)	0.6 (0.3–1.0)	0.6 (0.3–1.2)	0.6 (0.3–1.3)	0.6 (0.3–1.1)

*OR* odds ratio, *CI* confidence interval. Bold texts denotes a > 10% change in the OR from the unadjusted value

### Multivariable adjusted associations with oral HPV infection

We included each variable that was significant in bivariable analyses in a multivariable model along with the variables that were diagnosed as potential confounders (Table 5). In this model, CD4+ level continued to show an association with oral HPV when < 200 cells/uL compared with 500+ (OR: 2.9 [95% CI 1.0–9.1]). Reporting chewing tobacco ‘regularly’ also continued to show a protective effect (OR: 0.2 [95% CI 0.1–0.6]) in adjusted analyses as did performing oral sex on a woman (OR: 0.4 [95% CI 0.2–0.9]). When adjusted for the other factors, engaging in oral-anal contact was no longer significantly associated with oral HPV infection. While not significant in bivariable analyses, when the number of vaginal sex partners was included in the multivariable model, having 1–4 vaginal sex partners increased the odds of having oral HPV infection (OR: 2.7 [95% CI 1.1–6.5]), although the comparisons between having no partners, having 5–39 partners, and having 40+ partners were not significantly associated with oral HPV infection.

**Table 5:** Multivariable adjusted associations with oral HPV infection among HIV-positive Indian MSM

Characteristic	OR (95% CI)^a^	***P***-value
Age		
18–25	1.0	
26–35	1.5 (0.3–10.2)	0.5603
35+	2.4 (0.4–14.6)	0.3391
Income (rupees per year)		
> 2000	1.0	
2000–4999	1.0 (0.4–2.5)	0.9952
> 5000	0.9 (0.3–1.5)	0.8091
Married	0.6 (0.3–1.5)	0.2933
CD4+ level		
< 200	1.0	
200–500	2.0 (0.8–4.7)	0.1204
500+	2.9 (1.0–9.1)	0.0611
Chews tobacco regularly	0.2 (0.1–0.6)	0.0044
Performed oral sex on women, lifetime	0.4 (0.2–0.9)	0.0268
Ever engaged in oral-anal contact, lifetime	0.6 (0.2–1.4)	0.2138
Number of vaginal sex partners, lifetime		
0	1.0	
1–4	2.7 (1.1–6.5)	0.0277
5+	1.7 (0.6–4.6)	0.3275

^a^ OR, odds ratio and 95% CI, confidence interval from logistic regression multivariable model

## Discussion

This is the first report of the prevalence of oral HPV infection among Indian HIV-positive MSM. Our participants had a similar prevalence of any oral HPV infection compared with other HIV-positive populations, with previous estimates ranging from 14 to 45% [40, 41, 53]. However, unlike other reports of oral HPV among HIV-positive MSM, our participants had fewer oncogenic HPV types that are typically found in the anogenital tract. Only 6% of our participants had one of the 39 types for which we tested, only 2.4% had an oncogenic anogenital HPV type and none had HPV 16. Previous studies have found a prevalence between 11 and 20% of oncogenic HPV types and 7–10% HPV 16 in their study populations [40, 53]. Using the same HPV detection methods, our study population had high levels of penile (48%) [52] and anal (95%) [51] HPV infection, comparable to other populations of HIV-infected MSM. These same methods were also used in a recent study of oral HPV infection among MSM in San Francisco that reported a 30% prevalence of oral HPV with an 11% prevalence of oncogenic HPV types [54].

Although no studies of oral cancer among HIV-positve men in India have occurred to date, studies of HIV-negative men and women indicate that HPV does play a role in Indian oral and oropharyngeal cancers in India. The prevalence of HPV-16, for example, in biopsies of oral and oropharyngeal cancers of Indian HIV-negative men and women ranges from 0% [55, 56] to over 30% [13, 33, 57, 58] showing that oral HPV-16 infection may be important in Indian oral cancers as it is in other populations. However, the association between HPV and oral cancers has not been as well characterized in India as in other countries and the prevalence of other risk factors for oral cancers in India is high.

Another difference between our results and previously reported results was that individuals who reported chewing tobacco regularly had a lower prevalence of oral HPV infection than those who reported that they did not regularly chew tobacco and this association remained significant in multivariable adjusted analyses. Chewing tobacco has not been associated with oral HPV infection in previous studies, however it has been consistently associated with oral cancers [59, 60] and oral pre-cancerous lesions [61, 62]. Several studies have described two separate pathways to oral and oropharyngeal cancers; one that includes tobacco products and another that includes oral HPV infection [23, 63–66]. Studies that have examined effect modification between tobacco products and oral HPV have had conflicting results [67, 68]. It is possible that while chewing tobacco is associated with cancers of the oral cavity, it is not associated with cancers that are associated with HPV infection. The mechanism of protection provided by chewing tobacco in this case is not clear. It may be that the smokeless tobacco itself or another component in the chewing tobacco mixture may interfere with oral HPV infection. Also, in our study we did not clarify a difference between chewing tobacco and chewing betel quid (areca nut). Indian betel quid, a combination of betel leaf, smokeless tobacco, areca nut and lime paste (a mixture of calcium and hydroxide-calcium carbonate), has itself been associated with oral cancers [11–13]. It is possible, however, that one of the components of betel quid interferes with oral HPV infection. Finally, we cannot exclude the possibility that one or more components of chewing tobacco in the oral specimens interfered with detection of HPV DNA in our typing assay.

Two other factors showed protective effects in our analyses that were different from what has been shown in the literature [69], i.e. oral-anal contact (rimming) and having oral sex with women. These associations did not dissipate when adjusted for marital status, number of female vaginal sex partners or number of oral sex partners. However, in our fully adjusted model (that included both chewing tobacco and oral sex with women), oral-anal contact was no longer significant. We found a limited number of anogenital HPV types in our oral samples in our study and sexual risk factors may not be as important in this population. This implies that non-sexual routes of exposure may be more significant to the development of oral HPV infection than sexual ones in this population. Although this does not explain the protective effect we found with oral-anal contact and oral sex with women, it may explain why they were not found to be risk factors. It may be that that these sexual behaviors are markers of behaviors that participants do not engage in, or engage in less often. Focus group discussions and key informant interviews should precede any future study on oral HPV infection with HIV-positive MSM in India.

In contrast to the behavioral data, the results collected with a biological markers of HIV disease status were consistent with data from Western populations. Among our study participants, having a lower CD4+ level was associated with a higher prevalence of oral HPV infection. Studies of Western HIV-positive MSM have also found that lower CD4+ level was associated with higher risk of oral HPV infection in men [40, 41] and a number of studies have also seen this association among HIV-positive women [41, 48]. This is also consistent with the relationship seen with anal and cervical HPV infection where lower CD4+ levels are associated with increased incidence and prevalence of HPV infection and HPV-associated disease [49, 51, 70–74].

Our study had a few limitations. It was designed as a cross-sectional study because it was the first study of oral HPV among HIV-positive men in India. Additionally, we did not have a random sample of Indian HIV-positive MSM and the results may not be generalizable to all HIV-positive Indian MSM but only Indian MSM living in urban centers with access to medical services. However, MSM behavior and HIV-positive status are both highly stigmatized in India, and it is unlikely that other sampling strategies would have yielded a more representative sample while ensuring participant confidentiality.

## Conclusion

Although our study has confirmed that Indian HIV-positive MSM have a similar prevalence of oral HPV infection as in previous reports from other parts of the world, they have fewer anogenital HPV types, particularly oncogenic HPV types, detectable in the mouth. This is in stark contrast to what we found for penile [52] and anal HPV infection [51] in this same population which had very similar distribution of oncogenic anogenital HPV types to other HIV-positive populations in other locations. Indian HIV-positive MSM also differ from Western MSM in lifestyle, sexual history, and cultural practices and thus may have different risk factors for oral HPV infection than they do for penile and anal HPV infections. Although oral and oropharyngeal cancer are very important in India, oral HPV infection may not be as important in this population as it is in previously studied populations. Studies of oral cancer among HIV-positive men would help elucidate the role of HPV in oral and oropharyngeal cancers in India.

## Supplementary Information

**Additional file 1: Supplementary File 1.** Supplement 1 - Questionnaire, English language version of the Questionnaire used in the study.

## Data Availability

The datasets used and/or analyzed during the current study are available from the corresponding author on reasonable request.

## Abbreviations

ANOVA: Analysis of variance
ART: Antiretroviral therapy
CD4: Cluster of differentiation 4
CI: Confidence interval
CMC: Christian Medical College, Vellore, India
DNA: Deoxyribonucleic acid
HIV/AIDS: Human immunodeficiency virus/acquired immunodeficiency syndrome
HPV: Human papillomavirus
HT: Humsafar Trust, Mumbai, India
L1: Major capsid protein
NGO: Non-governmental organization
MSM: Men who have sex with men
OR: Odds ratios
OSCC: Oral squamous cell carcinoma
PCR: Polymerase chain reaction
VL: Viral load
