# Supplementary material for: Prevalence of oral human papillomavirus infection among Indian HIV-positive men who have sex with men: a cross-sectional study
Source: BMC Infect Dis. 2021 Jul 12;21:675. doi: 10.1186/s12879-021-06301-6 (PMC8274002; doi:10.1186/s12879-021-06301-6)
Supplement: Supplementary file 1 — Additional file 1: Supplementary File 1. Supplement 1 - Questionnaire, English language version of the Questionnaire used in the study. [file 12879_2021_6301_MOESM1_ESM.pdf]

HPV IN INDIAN MEN (HIIM) RESEARCH GROUP

MUMBAI AND VELLORE HPV STUDY
BASELINE QUESTIONNAIRE

Clinic Site: ☐ *HT* ☐ *CMC* *Participant ID#:*________________________

A: Interview Information

Instructions, notes, and prompts for interviewer are included throughout in italics or [Brackets]

1. Date of Interview

____/____/____*dd/mmm/yyyy*

2. Interviewer Initials

☐☐

3. Language of Interview

English ☐ 1
Hindi ☐ 2
Tamil ☐ 3

4. How did you hear about this study?

Prompt HT: "Does your friend work at Humsafar" if the answer is yes, then select option 1.

Outreach worker ☐ 1
Referred by friend ☐ 2
Referred by clinician ☐ 3
Referred by GLOW ☐ 4
Referred by ICWO ☐ 5
Referred by other NGO ☐ 6
Other: ☐ 7
If other please specify
____

Decline ☐ 99

B: Demographics

Interviewer, please read the following to the participant:

Thank you for agreeing to participate in our study. This interview asks many questions. Some are personal about your life, and your health, and your sex partners. All your answers are strictly confidential and, it is very important for us that you try to answer all the questions as openly as possible, even if they are a little uncomfortable or embarrassing. This information will be important in understanding how to develop programs to promote sexual health for men in your community. If you don't understand any of the questions, please ask and I will try to help explain them.

I would like to start by asking you some general questions:

5. Where do you live (for most of the year)?

____________________ *city*

____________________ *state*

Decline ☐ 99

6. Have you traveled out of ____________(*insert answer from question 5*) for business or pleasure in the last 6 months?

Yes ☐ 1 No ☐ 0
If no skip to Q7

Decline ☐ 99

***HPV IN INDIA MEN (HIIM) Research Group** – A Collaboration among the University of California, San Francisco (UCSF), Christian Medical College, Vellore, Humsafar Trust Mumbai, And The AIDS Malignancy Consortium (AMC). Correspondence to alexandra.hernandez@ucsf.edu.*

	a. Where did you travel?	____________________ *city*<br>___________________ *state*<br>Decline ☐ 99
**7.**	**What is your native place (village)?**<br>*NOTE: If the participant only knows the state write "Unknown" in city/district.*	____________________<br>*city/district*<br>___________________ *state*<br>Decline ☐ 99
**8.**	**What is your marital status?**	Married ☐ 1<br>Single/Never married ☐ 2<br>Divorced ☐ 3<br>Separated ☐ 4<br>Widowed ☐ 5<br>Decline ☐ 99
**9.**	**Whom do you live with?**<br>*PROMPTS::*<br>*"A Male Partner is a boyfriend or a steady male partner that you are living with"*<br>*NOTE: If the participant is living with an unmarried female partner, check Other and specify.*	With wife and/or children ☐ 1<br>With parents and/or wife and/or children ☐ 2<br>With male partner ☐ 3<br>With non-partner male friends ☐ 4<br>Alone ☐ 5<br>With Guru ☐ 6<br>Paying guest/Hostel ☐ 7<br>Other ☐ 8<br>*If other please specify* ____________________<br>Decline ☐ 99
**10.**	**Are you currently working?**<br>*PROMPT: If 'no' first ask "Do you have any source of income" or "How do you earn you living" If the answer fits any category below, check yes and ask 10a.*	Yes ☐ 1 No ☐ 0<br>*If no skip to Q11*<br>Decline ☐ 99
	a. What is your primary occupation (What work do you do)?<br>*NOTE: Chose only one option.*	Company/factory worker ☐ 1<br>Day laborer ☐ 2<br>Student ☐ 3<br>Government worker ☐ 4<br>Store/stall vendor ☐ 5<br>Truck driver ☐ 6<br>Taxi/rickshaw driver ☐ 7<br>Waiter/food service/hotel ☐ 8<br>Clerical worker ☐ 9<br>Artisan ☐ 10<br>Sex worker ☐ 11<br>Other ☐ 12<br>*If other please specify*<br>____________________<br>Decline ☐ 99

***HPV IN INDIA MEN (HIIM) Research Group** – A Collaboration among the University of California, San Francisco (UCSF), Christian Medical College, Vellore, Humsafar Trust Mumbai, And The AIDS Malignancy Consortium (AMC). Correspondence to alexandra.hernandez@ucsf.edu.*

11. What is your monthly income from all sources? _________ INR

Decline ☐ 99

12. What was the highest grade or year of school you completed?

None ☐ 1
School (1- 10th) ☐ 2
College (11-12) ☐ 3
Graduation (13-15) ☐ 4
Post Grad/Professional ☐ 5

Decline ☐ 99

13. What is your religion?

Hindu ☐ 1
Muslim ☐ 2
Christian ☐ 3
Sikh ☐ 4
Buddhist ☐ 5
Jain ☐ 6
None ☐ 7
Other: ☐ 8
If other please specify ________________________

Decline ☐ 99

C: Smoking, Alcohol, Drugs

14. Have you smoked more than 100 cigarettes/beedis until now? Yes ☐ 1 No ☐ 0
If no skip to Q15

Decline ☐ 99

a. Do you currently smoke? Yes ☐ 1 No ☐ 0
If no skip to Q15

Decline ☐ 99

b. How many years total have you smoked cigarettes? _____________ *years*
PROMPT: "How old where you when you started smoking"

c. On average do you smoke a pack or more each day? Yes ☐ 1 No ☐ 0
If no skip to Q15

Decline ☐ 99

d. On average how many packs of cigarettes do you smoke each day? __________ *number of packs*

Decline ☐ 99

15. Do you chew tobacco regularly? Yes ☐ 1 No ☐ 0
PROMPT: "Chewing tobacco is also called Pan Parag, Jaritha, Gutka, or Hans"
If no skip to Q16

Decline ☐ 99

HPV IN INDIA MEN (HIIM) Research Group *– A Collaboration among the University of California, San Francisco (UCSF), Christian Medical College, Vellore, Humsafar Trust Mumbai, And The AIDS Malignancy Consortium (AMC). Correspondence to alexandra.hernandez@ucsf.edu.*

	a. On an average WEEK, how often do you chew tobacco?<br><br>*NOTE: If the participant responds Almost never, 1 every 2 week, or 1 a month etc, check "<1day/week"*<br>*If the participant responds, Always, or almost every day check "6-7 days/week"*	< 1 day/week ☐ 1<br>1-2 days/week ☐ 2<br>3-5 days/week ☐ 3<br>6-7 days/week ☐ 4<br><br>Decline ☐ 99
**16.**	**Have you ever consumed any alcoholic beverage, such as beer, wine, country made liquor (arrack) or other liquor?**	Yes ☐ 1 No ☐ 0<br>*If no skip to Q17*<br><br>Decline ☐ 99
	a. During the past <u>6 months</u> did you drink any alcoholic beverages?	Yes ☐ 1 No ☐ 0<br>*If no skip to Q17*<br><br>Decline ☐ 99
	b. During the past <u>6 months</u> on average, how many days per week did you drink?	< 1 day ☐ 1<br>1-2 days ☐ 2<br>3-5 days ☐ 3<br>6-7 days ☐ 4<br><br>Decline ☐ 99
	c. If we define a drink as 1 beer or a pint or a quarter of liquor: During the past <u>6 months,</u> on average in one sitting, how many drinks did you have?<br><br>*PROMPT: "Please give me your best guess or estimate"*	___________ *# drink*<br><br>Decline ☐ 99
	d. During the past <u>6 months</u>, when you drank, how often did you become "drunk" or inebriated?	Never ☐ 1<br>< ½ the time ☐ 2<br>About ½ the time ☐ 3<br>> ½ the time ☐ 4<br>Always ☐ 5<br><br>Decline ☐ 99
**17.**	**Have you ever used any drugs for pleasure?**<br><br>*PROMPT: Drugs can include bhang/ ganja, heroin, brown sugar, stimulants, cocaine, Hallucinogens, Inhalants, amphetamines, or tranquilozers"*<br><br>*NOTE: If the participant mentions any of the drugs listed below as an example, check 'yes' and continue.*	Yes ☐ 1 No ☐ 0<br>*If no skip to Q26*<br>Decline ☐ 99
**18.**	**Have you ever used marijuana (bhang/ ganja/charas)?**	Yes ☐ 1 No ☐ 0<br>*If no skip to Q19*<br>Decline ☐ 99
	a. How many times total until now have you used marijuana (bhang/ ganja)?	1-9 ☐ 1<br>10-100 ☐ 2<br>101-500 ☐ 3<br>500+ ☐ 4<br><br>Decline ☐ 99

HPV IN INDIA MEN (HIIM) Research Group – A Collaboration among the University of California, San Francisco (UCSF), Christian Medical College, Vellore, Humsafar Trust Mumbai, And The AIDS Malignancy Consortium (AMC). Correspondence to alexandra.hernandez@ucsf.edu.

b.	During the past <u>6 months</u>, did you use marijuana (bhang/ ganja)?	Yes ☐ 1 No ☐ 0<br>*If no skip to Q19*<br>Decline ☐ 99
c.	During the past <u>6 months</u>, many days per week did you use marijuana (bhang/ ganja)?	< 1 day/week ☐ 1<br>1-2 days/week ☐ 2<br>3-5 days/week ☐ 3<br>6-7 days/week ☐ 4<br>Decline ☐ 99
**19.**	**Have you ever injected heroin into your vein or skin, for pleasure?**	Yes ☐ 1 No ☐ 0<br>*If no skip to Q20*<br>Decline ☐ 99
a.	How many times total until now have you injected heroin?	1-9 ☐ 1<br>10-100 ☐ 2<br>101-500 ☐ 3<br>500+ ☐ 4<br>Decline ☐ 99
b.	During the past <u>6 months</u>, did you inject heroin?	Yes ☐ 1 No ☐ 0<br>*If no skip to Q20*<br>Decline ☐ 99
c.	During the past <u>6 months</u>, how many days per week did you did you inject heroin?	< 1 day/week ☐ 1<br>1-2 days/week ☐ 2<br>3-5 days/week ☐ 3<br>6-7 days/week ☐ 4<br>Decline ☐ 99
**20.**	**Have you ever snorted or injected Brown Sugar into your vein or skin, for pleasure?**	Yes ☐ 1 No ☐ 0<br>*If no skip to Q21*<br>Decline ☐ 99
a.	How many times total until now have you injected Brown Sugar?	1-9 ☐ 1<br>10-100 ☐ 2<br>101-500 ☐ 3<br>500+ ☐ 4<br>Decline ☐ 99
b.	During the past <u>6 months</u>, did you inject Brown Sugar?	Yes ☐ 1 No ☐ 0<br>*If no skip to Q21*<br>Decline ☐ 99
c.	During the past <u>6 months</u>, how many days per week did you inject Brown Sugar?	< 1 day/week ☐ 1<br>1-2 days/week ☐ 2<br>3-5 days/week ☐ 3<br>6-7 days/week ☐ 4<br>Decline ☐ 99

HPV IN INDIA MEN (HIIM) Research Group *– A Collaboration among the University of California, San Francisco (UCSF), Christian Medical College, Vellore, Humsafar Trust Mumbai, And The AIDS Malignancy Consortium (AMC). Correspondence to alexandra.hernandez@ucsf.edu.*

**21.**	**Have you ever used Stimulants (amphetamines, speed, crystal meth, rush , Dexedrine Ritalin, diet pills, Viagra, vitaex gold)?**	Yes ☐ 1 No ☐ 0<br>*If no skip to Q22*<br>Decline ☐ 99
a.	How many times total until now have you used Stimulants?	1-9 ☐ 1<br>10-100 ☐ 2<br>101-500 ☐ 3<br>500+ ☐ 4<br>Decline ☐ 99
b.	During the past <u>6 months</u>, did you use Stimulants?	Yes ☐ 1 No ☐ 0<br>*If no skip to Q22*<br>Decline ☐ 99
c.	During the past <u>6 months</u>, how many days per week did you use Stimulants?	< 1 day/week ☐ 1<br>1-2 days/week ☐ 2<br>3-5 days/week ☐ 3<br>6-7 days/week ☐ 4<br>Decline ☐ 99
**22.**	**Have you ever used Cocaine?**	Yes ☐ 1 No ☐ 0<br>*If no skip to Q23*<br>Decline ☐ 99
a.	How many times total until know have you used Cocaine?	1-9 ☐ 1<br>10-100 ☐ 2<br>101-500 ☐ 3<br>500+ ☐ 4<br>Decline ☐ 99
b.	During the past <u>6 months</u>, did you use Cocaine?	Yes ☐ 1 No ☐ 0<br>*If no skip to Q23*<br>Decline ☐ 99
c.	During the past <u>6 months</u>, how many days per week did you use Cocaine?	< 1 day/week ☐ 1<br>1-2 days/week ☐ 2<br>3-5 days/week ☐ 3<br>6-7 days/week ☐ 4<br>Decline ☐ 99
**23.**	**Have you ever used Hallucinogens (LSD, acid, mescaline, peyote, PCP, ecstasy, MDA or MDMA)?**	Yes ☐ 1 No ☐ 0<br>*If no skip to Q24*<br>Decline ☐ 99

***HPV IN INDIA MEN (HIIM) Research Group** – A Collaboration among the University of California, San Francisco (UCSF), Christian Medical College, Vellore, Humsafar Trust Mumbai, And The AIDS Malignancy Consortium (AMC). Correspondence to alexandra.hernandez@ucsf.edu.*

---

a. How many times total until now have you used Hallucinogens?

1-9 ☐ 1
10-100 ☐ 2
101-500 ☐ 3
500+ ☐ 4

Decline ☐ 99

---

b. During the past <u>6 months</u>, did you use Hallucinogens?

Yes ☐ 1 No ☐ 0
If no skip to Q24

Decline ☐ 99

---

c. During the past <u>6 months</u>, how many days per week did you use Hallucinogens?

< 1 day/week ☐ 1
1-2 days/week ☐ 2
3-5 days/week ☐ 3
6-7 days/week ☐ 4

Decline ☐ 99

---

24. Have you ever used Inhalants (glue, ethyl chloride, nitrous oxide, amyl or butyl nitrate, poppers, korex, whitener)?

Yes ☐ 1 No ☐ 0
If no skip to Q25

Decline ☐ 99

---

a. How many times total until now have you used Inhalants?

1-9 ☐ 1
10-100 ☐ 2
101-500 ☐ 3
500+ ☐ 4

Decline ☐ 99

---

b. During the past <u>6 months</u>, did you use Inhalants?

Yes ☐ 1 No ☐ 0
If no skip to Q25

Decline ☐ 99

---

c. During the past <u>6 months</u>, how many days per week did you use Inhalants?

< 1 day/week ☐ 1
1-2 days/week ☐ 2
3-5 days/week ☐ 3
6-7 days/week ☐ 4

Decline ☐ 99

---

25. Have you ever used Tranquilizers (Quaalude, seconal, valium, xanax Librium, barbiturates)?

Yes ☐ 1 No ☐ 0
If no skip to Q26

Decline ☐ 99

---

a. How many times total until now have you used Tranquilizers?

1-9 ☐ 1
10-100 ☐ 2
101-500 ☐ 3
500+ ☐ 4

Decline ☐ 99

HPV IN INDIA MEN (HIIM) Research Group – A Collaboration among the University of California, San Francisco (UCSF), Christian Medical College, Vellore, Humsafar Trust Mumbai, And The AIDS Malignancy Consortium (AMC). Correspondence to alexandra.hernandez@ucsf.edu.

b. During the past <u>6 months</u>, did you use Tranquilizers?	Yes ☐ 1 No ☐ 0<br>*If no skip to Q26*<br>Decline ☐ 99
c. During the past <u>6 months</u>, how many days per week did you use Tranquilizers?	< 1 day/week ☐ 1<br>1-2 days/week ☐ 2<br>3-5 days/week ☐ 3<br>6-7 days/week ☐ 4<br>Decline ☐ 99
**D: HIV and HAART**	
**26. When did you first have a positive HIV test?**<br>*PROMT: If they do not remember please say "Please give me your best guess as to the month and year of your first positive HIV test."*	____/____*mmm/yyyy*<br>Decline ☐ 99
**27. Have you ever had a CD4+ count test?**<br>*PROMT: "A blood test the perform after you have had your HIV+ diagnosis to check you HIV disease progression"*	Yes ☐ 1 No ☐ 0<br>*If no skip to Q28*<br>Decline ☐ 99
a. What was your lowest CD4+ count?	________________<br>Don't Know ☐ 88
**28. Have you ever taken any prescription medication to lower your HIV viral load (Highly Active Antiretroviral Therapy (HAART) or ART)?**	Yes ☐ 1 No ☐ 0<br>*If no skip to Q29*<br>Decline ☐ 99
a. Are you currently on HAART/ART?	Yes ☐ 1 No ☐ 0<br>*If no skip to Q29*<br>Decline ☐ 99
b. When did you first start taking HAART/ART?	____/____*mmm/yyyyy*<br>Decline ☐ 99
c. Where are you receiving your HAART/ART medication?	Government Hospital ☐ 1<br>MSF ☐ 2<br>NGO/Private Charity ☐ 3<br>Private Doctor/Clinic ☐ 4<br>Other ☐ 5<br>Please specify:_______________
d. Have you ever stopped taking HAART?	Yes ☐ 1 No ☐ 0<br>Decline ☐ 99

HPV IN INDIA MEN (HIIM) Research Group – A Collaboration among the University of California, San Francisco (UCSF), Christian Medical College, Vellore, Humsafar Trust Mumbai, And The AIDS Malignancy Consortium (AMC). Correspondence to alexandra.hernandez@ucsf.edu.

e. How many months have you been on your current HAART regime?

______ *months*

Decline ☐ 99

f. Do you know the name or any of the drugs that are in your HAART/ART regime?

Yes ☐ 1 No ☐ 0

If no skip to Q29

Decline ☐ 99

g. Please tell me if you are taking any of the following drugs called nucleoside or nucleotide reverse transcriptase inhibitors (NRTI) ?

Abacavir, Ziagen ☐ 1
3TC, Lamivudine, Epivir ☐ 2
d4T, Stavudine, Zerit ☐ 3
ddC, Zalcitabine, Hivid ☐ 4
ddT, Didanosine, Videx ☐ 5
AZT, Zidovudine,Retrovir ☐ 6
Tenofovir, Viread ☐ 7
Emtricitabine ☐ 8

Don't Know ☐ 88

h. Please tell me if you are taking any of the following drugs called non-nucleoside or nucleotide reverse transcriptase inhibitors (NNRTI) ?

Delavirdine,Rescriptor,DLV ☐ 1
Efavirenz, Sustiva ☐ 2
Nevirapine,Viramune,NVP ☐ 3

Don't Know ☐ 88

i. Please tell me if you are taking any of the following drugs called protease inhibitors(PI) ?

Invirase, Saquinavir, SQV ☐ 1
Norvir, Ritonavir, RTV ☐ 2
Crixivan, Indinavir, IDV ☐ 3
Viracept, Nelfinavir, NLV e ☐ 4
Amprenavire, Agenerase ☐ 5
Lopinavir, Kaletra ☐ 6

Don't Know ☐ 88

E: Circumcision

29. Are you circumcised?

Yes ☐ 1 No ☐ 0
If no skip to Q30

Decline ☐ 99

a. How old were you when you were circumcised?

At Birth Yes ☐ 1 No ☐ 0 Don't Know ☐ 88
If no enter age and skip to Q30

_______ *age in years*

F: Genital Hygiene

30. In general do you push back your foreskin when you wash your penis?

Yes ☐ 1
No ☐ 2
Don't Know ☐ 3
Decline ☐ 99

HPV IN INDIA MEN (HIIM) Research Group *– A Collaboration among the University of California, San Francisco (UCSF), Christian Medical College, Vellore, Humsafar Trust Mumbai, And The AIDS Malignancy Consortium (AMC). Correspondence to alexandra.hernandez@ucsf.edu.*

**31.**	**In general do you notice smegma during the times you push back your foreskin?**	Yes ☐ 1<br>No ☐ 2<br>Don't Know ☐ 3<br>Decline ☐ 99
**32.**	**Do you wash your penis after insertive anal intercourse?**	Yes ☐ 1<br>No ☐ 0<br>No insertive intercourse☐ 2<br><br>*If 'no' skip to Q 33*<br><br>Decline ☐ 99
	a. How often do you wash your penis after anal intercourse?	Always ☐ 1<br>Sometimes ☐ 2<br>Rarely ☐ 3<br>Never ☐ 4<br><br>Decline ☐ 99

G: History of Warts

		a. Have you ever had:	b .How many times have you had _____?	c. Year you first had _____?	d. Year you last had _____?
**33.**	**Anal Warts**<br><br>*PROMPT:*<br>*"External growths on or around the anus"*	Yes ☐ 1<br>No ☐ 0<br>Decline ☐ 99<br>*If no skip to Q34*	☐☐	☐☐☐☐	☐☐☐☐
**34.**	**Penile Warts**<br><br>*PROMPT:*<br>*"External growths on or around the penis"*	Yes ☐ 1<br>No ☐ 0<br>Decline ☐ 99<br>*If no skip to Q35*	☐☐	☐☐☐☐	☐☐☐☐
**35.**	**Warts in the mouth**<br><br>*PROMPT:*<br>*"External growths in or on the mouth"*	Yes ☐ 1<br>No ☐ 0<br>Decline ☐ 99<br>*If no skip to Q36*	☐☐	☐☐☐☐	☐☐☐☐

I: Rectal Area Problems :

Next are some questions about other conditions or problems you may have had with your rectal area.

	a. Have you ever had:	b. *Within the last 12 months, how many total weeks have you had_____?*	c. Between 1& 5 years ago, how many total weeks did you have _____? *(If none enter 00)*	d. What year did you first have _____?	e. What year did you last have _____?
**36.** **Inflamed hemorrhoids or piles?**	Yes ☐ 1<br>No ☐ 0<br>Decline ☐ 99<br>*If no skip to Q37*	☐☐	☐☐	☐☐☐☐	☐☐☐☐

	a. Have you ever used [substance] regularly (6 times during a 6 month period):	b. What year did you first use _____ regularly?	c. What year did you last use _____ regularly?	d. During that period of regular use how often did you use them _____?
**37.** **Laxatives?**<br>*PROMPTS: "Examples are: Dulcolax, Buryolax, or stool softeners"*	Yes ☐ 1<br>No ☐ 0<br>Decline ☐ 99<br>*If no skip to Q38*			1 time/month or less ☐ 1<br>2-7 times/month ☐ 2<br>8-19 times/month ☐ 3<br>20+ times/month ☐ 4

***HPV IN INDIA MEN (HIIM) Research Group** – A Collaboration among the University of California, San Francisco (UCSF), Christian Medical College, Vellore, Humsafar Trust Mumbai, And The AIDS Malignancy Consortium (AMC). Correspondence to alexandra.hernandez@ucsf.edu.*

J: Sexual behavior

Please read the following to the participant:

In this last section of the questionnaire I have some questions about sexual practices you may have engaged in over the years. These questions are included because certain health problems may be affected by the way sexual relations are performed and also by the health or sexual partners.

Male Partner

Please read the following to the participant:

When I say 'Male Partner' I mean any male or transgender partner who you have had sex with.

38. Have you ever had any type of sexual contact with a male? Yes ☐ 1 No ☐ 0

NOTE: If the answer is no please review eligibility form to confirm eligibility. Decline ☐ 99

a. How old were you when you first had sexual contact with a male? ______ *age*

b. What is the number of male partners you have had until now? This would include any type of sexual activity with any male partner.

- 1-10 ☐ 1
- 11-50 ☐ 2
- 51-100 ☐ 3
- 101-200 ☐ 4
- 201-500 ☐ 5
- 501-1000 ☐ 6
- 1000+ ☐ 7
- Decline ☐ 99

39. How many male sexual partners have you had in the past 6 months?

NOTE: If '0' please review eligibility form to confirm eligibility.

- 0 ☐ 0
- 1 ☐ 1
- 2-4 ☐ 2
- 5-14 ☐ 3
- 15-29 ☐ 4
- 30-49 ☐ 5
- 50-99 ☐ 6
- 100+ ☐ 7
- Decline ☐ 99

40. How many male sexual partners have you had in the past 30 days?

- 0 ☐ 0
- 1 ☐ 1
- 2-4 ☐ 2
- 5-14 ☐ 3
- 15-29 ☐ 4
- 30-49 ☐ 5
- 50-99 ☐ 6
- 100+ ☐ 7
- Decline ☐ 99

Male Partner - Receptive

**41.**	**Have you ever had receptive anal intercourse with a male? Receptive anal intercourse is your male sexual partner inserting his penis into your anus/rectum.**	Yes ☐ 1 No ☐ 0<br>*If no skip to Q*<br>Decline ☐ 99
	a. How old were you when you first had receptive anal intercourse with at man?	______ *age*
	b. What is the number of male partners you have had receptive anal intercourse with until now?	1-10 ☐ 1<br>11-50 ☐ 2<br>51-100 ☐ 3<br>101-200 ☐ 4<br>201-500 ☐ 5<br>501-1000 ☐ 6<br>1000+ ☐ 7<br><br>Decline ☐ 99
	c. Please tell me on average, until now, how often you have receptive anal intercourse?	Never ☐ 1<br>1 in a month or less ☐ 2<br>1 time per week ☐ 3<br>3-4 per week ☐ 4<br>5-10 per week ☐ 5<br>11-20 per week ☐ 6<br>20+ per week ☐ 7<br><br>Decline ☐ 99
	d. On average, until now, how often has your partner used a condom during receptive anal intercourse?	Never ☐ 1<br>Occasionally ☐ 2<br>Half the time ☐ 3<br>Almost Always ☐ 4<br>Always ☐ 5<br><br>Decline ☐ 99
**42.**	**How many male sexual partners have you had receptive anal intercourse in the past 6 months.**	0 ☐ 0<br>1 ☐ 1<br>2-4 ☐ 2<br>5-14 ☐ 3<br>15-29 ☐ 4<br>30-49 ☐ 5<br>50-99 ☐ 6<br>100+ ☐ 7<br><br>Decline ☐ 99<br>*If 0 skip to Q44*

***HPV IN INDIA MEN (HIIM) Research Group** – A Collaboration among the University of California, San Francisco (UCSF), Christian Medical College, Vellore, Humsafar Trust Mumbai, And The AIDS Malignancy Consortium (AMC). Correspondence to alexandra.hernandez@ucsf.edu.*

a.	How often did you have receptive anal intercourse in the past 6 months?	Never ☐ 1<br>1 in a month or less ☐ 2<br>1 time per week ☐ 3<br>3-4 per week ☐ 4<br>5-10 per week ☐ 5<br>11-20 per week ☐ 6<br>20+ per week ☐ 7<br><br>Decline ☐ 99
b.	On average, in the past 6 months, how often have you used a condom during receptive anal intercourse?	Never ☐ 1<br>Occasionally ☐ 2<br>Half the time ☐ 3<br>Almost Always ☐ 4<br>Always ☐ 5<br><br>Decline ☐ 99
**43.**	**How many male sexual partners have you had receptive anal intercourse in the past 30 days?**	0 ☐ 0<br>1 ☐ 1<br>2-4 ☐ 2<br>5-14 ☐ 3<br>15-29 ☐ 4<br>30-49 ☐ 5<br>50-99 ☐ 6<br>100+ ☐ 7<br>*If 0 skip to Q44*<br><br>Decline ☐ 99
a.	How often did you have receptive anal intercourse in the past 30 days?	Never ☐ 1<br>1 in a month or less ☐ 2<br>1 time per week ☐ 3<br>3-4 per week ☐ 4<br>5-10 per week ☐ 5<br>11-20 per week ☐ 6<br>20+ per week ☐ 7<br><br>Decline ☐ 99
b.	On average, in the past 30 days, how often has your partner used a condom during receptive anal intercourse?	Never ☐ 1<br>Occasionally ☐ 2<br>Half the time ☐ 3<br>Almost Always ☐ 4<br>Always ☐ 5<br><br>Decline ☐ 99

Male Partner – Insertive

**44.**	**Have you ever had insertive anal intercourse with a male? Insertive anal intercourse is you inserting your penis into your male sexual partners anus/rectum.**	Yes ☐ 1 No ☐ 0<br>*If no skip to Q47*<br>Decline ☐ 99

HPV IN INDIA MEN (HIIM) Research Group – A Collaboration among the University of California, San Francisco (UCSF), Christian Medical College, Vellore, Humsafar Trust Mumbai, And The AIDS Malignancy Consortium (AMC). Correspondence to alexandra.hernandez@ucsf.edu.

a. How old were you when you first had insertive anal intercourse with a man? ______ *age*

Decline ☐ 99

b. What is the number of male partners you have insertive anal intercourse with until now?

1-10 ☐ 1
11-50 ☐ 2
51-100 ☐ 3
101-200 ☐ 4
201-500 ☐ 5
501-1000 ☐ 6
1000+ ☐ 7

Decline ☐ 99

e. Please tell me on average, until now, how often you have insertive anal intercourse?

Never ☐ 1
1 in a month or less ☐ 2
1 time per week ☐ 3
3-4 per week ☐ 4
5-10 per week ☐ 5
11-20 per week ☐ 6
20+ per week ☐ 7

Decline ☐ 99

c. On average, until now how often have you used a condom during Insertive anal intercourse?

Never ☐ 1
Occasionally ☐ 2
Half the time ☐ 3
Almost Always ☐ 4
Always ☐ 5

Decline ☐ 99

45. How many male sexual partners have you had insertive anal intercourse in the past 6 months?

0 ☐ 0
1 ☐ 1
2-4 ☐ 2
5-14 ☐ 3
15-29 ☐ 4
30-49 ☐ 5
50-99 ☐ 6
100+ ☐ 7

Decline ☐ 99
If 0 skip to 47

HPV IN INDIA MEN (HIIM) Research Group *– A Collaboration among the University of California, San Francisco (UCSF), Christian Medical College, Vellore, Humsafar Trust Mumbai, And The AIDS Malignancy Consortium (AMC). Correspondence to alexandra.hernandez@ucsf.edu.*

a.	How often did you have insertive anal intercourse in the past 6 months?	Never ☐ 1<br>1 in a month or less ☐ 2<br>1 time per week ☐ 3<br>3-4 per week ☐ 4<br>5-10 per week ☐ 5<br>11-20 per week ☐ 6<br>20+ per week ☐ 7<br><br>Decline ☐ 99
b.	On average, in the past 6 months, how often have you used a condom during Insertive anal intercourse?	Never ☐ 1<br>Occasionally ☐ 2<br>Half the time ☐ 3<br>Almost Always ☐ 4<br>Always ☐ 5<br><br>Decline ☐ 99
**46.**	**How many male sexual partners have you had insertive anal intercourse in the past 30 days?**	0 ☐ 0<br>1 ☐ 1<br>2-4 ☐ 2<br>5-14 ☐ 3<br>15-29 ☐ 4<br>30-49 ☐ 5<br>50-99 ☐ 6<br>100+ ☐ 7<br><br>Decline ☐ 99<br>*If 0 skip to Q47*
a.	How often did you have insertive anal intercourse in the past 30 days?	Never ☐ 1<br>1 in a month or less ☐ 2<br>1 time per week ☐ 3<br>3-4 per week ☐ 4<br>5-10 per week ☐ 5<br>11-20 per week ☐ 6<br>20+ per week ☐ 7<br><br>Decline ☐ 99
b.	On average, in the past 30 days, how often have you used a condom during Insertive anal intercourse?	Never ☐ 1<br>Occasionally ☐ 2<br>Half the time ☐ 3<br>Almost Always ☐ 4<br>Always ☐ 5<br><br>Decline ☐ 99

Male Partner – Receptive Oral Sex

HPV IN INDIA MEN (HIIM) Research Group – A Collaboration among the University of California, San Francisco (UCSF), Christian Medical College, Vellore, Humsafar Trust Mumbai, And The AIDS Malignancy Consortium (AMC). Correspondence to alexandra.hernandez@ucsf.edu.

47. Have you ever performed oral sex on a male? Oral sex would be a blow job or you sucking someone's penis.

Yes ☐ 1 No ☐ 0
If no skip to Q50

Decline ☐ 99

a. In your life, how many males have you performed oral sex on until now?

1-10 ☐ 1
11-50 ☐ 2
51-100 ☐ 3
101-200 ☐ 4
201-500 ☐ 5
501-1000 ☐ 6
1000+ ☐ 7

Decline ☐ 99

48. How many male sexual partners have you performed oral sex on in the past 6 months?

0 ☐ 0
1 ☐ 1
2-4 ☐ 2
5-14 ☐ 3
15-29 ☐ 4
30-49 ☐ 5
50-99 ☐ 6
100+ ☐ 7

Decline ☐ 99
If 0 skip toQ50

a. During the past 6 months, when performing oral sex, how often did your partner wear a condom?

Never ☐ 1
Occasionally ☐ 2
Half the time ☐ 3
Almost Always ☐ 4
Always ☐ 5

Decline ☐ 99

49. How many male sexual partners have you performed oral sex on in the past 30 days?

0 ☐ 0
1 ☐ 1
2-4 ☐ 2
5-14 ☐ 3
15-29 ☐ 4
30-49 ☐ 5
50-99 ☐ 6
100+ ☐ 7

Decline ☐ 99
If 0 skip to Q50

a. During the past 30 days, when performing oral sex, how often did your partner wear a condom?

Never ☐ 1
Occasionally ☐ 2
Half the time ☐ 3
Almost Always ☐ 4
Always ☐ 5

Decline ☐ 99

***HPV IN INDIA MEN (HIIM) Research Group** – A Collaboration among the University of California, San Francisco (UCSF), Christian Medical College, Vellore, Humsafar Trust Mumbai, And The AIDS Malignancy Consortium (AMC). Correspondence to alexandra.hernandez@ucsf.edu.*

Male Partner – Rimming

50. Have you ever engaged in rimming? Rimming is oral/anal contact as either a giving or receiving partner and can also be described as licking your male sexual partners anus or your male sexual partner licking your anus.

Yes ☐ 1 No ☐ 0
If no skip toQ51
Decline ☐ 99

a. What is the number of partners you have rimmed until now? (Your mouth on your partners anus or you licking your partners anus)

0 ☐ 0
1-10 ☐ 1
11-50 ☐ 2
51-100 ☐ 3
101-200 ☐ 4
201-500 ☐ 5
501-1000 ☐ 6
1000+ ☐ 7
Decline ☐ 99

b. What is the number of partners who have rimmed you until now? (Your partners mouth to your anus or your sexual partner licking your anus)

0 ☐ 0
1-10 ☐ 1
11-50 ☐ 2
51-100 ☐ 3
101-200 ☐ 4
201-500 ☐ 5
501-1000 ☐ 6
1000+ ☐ 7
Decline ☐ 99

Female Partner

51. Have you ever had any type of sexual contact with a female?

Yes ☐ 1 No ☐ 0
If no skip to Q61
Decline ☐ 99

52. Have you ever had vaginal intercourse with a female?

Yes ☐ 1 No ☐ 0
If no skip to Q55
Decline ☐ 99

a. How old were you when you first had vaginal intercourse with a female? ______ *age*

b. What is the number of female partners you have had vaginal intercourse with until now?

1-4 ☐ 1
5-9 ☐ 2
10-19 ☐ 3
20-39 ☐ 4
40+ ☐ 5
Decline ☐ 99

HPV IN INDIA MEN (HIIM) Research Group – A Collaboration among the University of California, San Francisco (UCSF), Christian Medical College, Vellore, Humsafar Trust Mumbai, And The AIDS Malignancy Consortium (AMC). Correspondence to alexandra.hernandez@ucsf.edu.

c. On average, until now how often have you used a condom during vaginal intercourse?

Never ☐ 1
Occasionally ☐ 2
Half the time ☐ 3
Almost Always ☐ 4
Always ☐ 5

Decline ☐ 99

53. How many Female sexual partners did you have vaginal intercourse with in the past 6 months?

0 ☐ 0
1 ☐ 1
2-4 ☐ 2
5-14 ☐ 3
15-29 ☐ 4
30-49 ☐ 5
50-99 ☐ 6
100+ ☐ 7

Decline ☐ 99
If 0 skip to61

a. How often did you have vaginal intercourse in the past 6 months?

Never ☐ 1
1/month or < ☐ 2
1/week ☐ 3
3-4 /week ☐ 4
5-10 /week ☐ 5
11-20 /week ☐ 6
20+/week ☐ 7

Decline ☐ 99

b. On average, in the past 6 months, how often have you used a condom during vaginal intercourse?

Never ☐ 1
Occasionally ☐ 2
Half the time ☐ 3
Almost Always ☐ 4
Always ☐ 5

Decline ☐ 99

54. How many Female sexual partners have you had vaginal intercourse with in the past 30 days?

0 ☐ 0
1 ☐ 1
2-4 ☐ 2
5-14 ☐ 3
15-29 ☐ 4
30-49 ☐ 5
50-99 ☐ 6
100+ ☐ 7

Decline ☐ 99
If 0 skip Q61

***HPV IN INDIA MEN (HIIM) Research Group** – A Collaboration among the University of California, San Francisco (UCSF), Christian Medical College, Vellore, Humsafar Trust Mumbai, And The AIDS Malignancy Consortium (AMC). Correspondence to alexandra.hernandez@ucsf.edu.*

a. How often did you have vaginal intercourse in the past 30 days?

Never ☐ 1
1/month or < ☐ 2
1/week ☐ 3
3-4 /week ☐ 4
5-10 /week ☐ 5
11-20 /week ☐ 6
20+/week ☐ 7

Decline ☐ 99

b. On average, in the past 30 days, how often have you used a condom during Vaginal intercourse with a female?

Never ☐ 1
Occasionally ☐ 2
Half the time ☐ 3
Almost Always ☐ 4
Always ☐ 5

Decline ☐ 99

Female Partner – Insertive anal

55. Have you ever had insertive anal intercourse with a woman (your penis inserted into her anus)?

Yes ☐ 1 No ☐ 0
If no skip to 58

Decline ☐ 99

a. How old were you when you first had insertive anal intercourse with at woman?

______ *age*

b. What is the number of female partners you have had insertive anal intercourse with a woman until now?

1-4 ☐ 1
5-9 ☐ 2
10-19 ☐ 3
20-39 ☐ 4
40+ ☐ 5

Decline ☐ 99

c. On average, until now how often have you used a condom during insertive anal intercourse with a woman?

Never ☐ 1
Occasionally ☐ 2
Half the time ☐ 3
Almost Always ☐ 4
Always ☐ 5

Decline ☐ 99

HPV IN INDIA MEN (HIIM) Research Group – A Collaboration among the University of California, San Francisco (UCSF), Christian Medical College, Vellore, Humsafar Trust Mumbai, And The AIDS Malignancy Consortium (AMC). Correspondence to alexandra.hernandez@ucsf.edu.

56. How many Female sexual partners did you have anal intercourse with in the past 6 months?

0 ☐ 0
1 ☐ 1
2-4 ☐ 2
5-14 ☐ 3
15-29 ☐ 4
30-49 ☐ 5
50-99 ☐ 6
100+ ☐ 7

Decline ☐ 99
If 0 skip to 58

a. How often did you have anal intercourse with a female in the past 6 months?

Never ☐ 1
1/month or < ☐ 2
1/week ☐ 3
3-4 /week ☐ 4
5-10 /week ☐ 5
11-20 /week ☐ 6
20+/week ☐ 7

Decline ☐ 99

b. On average, in the past 6 months, how often have you used a condom during anal intercourse with a female?

Never ☐ 1
Occasionally ☐ 2
Half the time ☐ 3
Almost Always ☐ 4
Always ☐ 5

Decline ☐ 99

57. How many Female sexual partners have you had anal intercourse with in the past 30 days?

0 ☐ 0
1 ☐ 1
2-4 ☐ 2
5-14 ☐ 3
15-29 ☐ 4
30-49 ☐ 5
50-99 ☐ 6
100+ ☐ 7

Decline ☐ 99
If 0 skip to Q58

a. How often did you have anal intercourse with a female in the past 30 days?

Never ☐ 1
1/month or < ☐ 2
1/week ☐ 3
3-4 /week ☐ 4
5-10 /week ☐ 5
11-20 /week ☐ 6
20+/week ☐ 7

Decline ☐ 99

***HPV IN INDIA MEN (HIIM) Research Group** – A Collaboration among the University of California, San Francisco (UCSF), Christian Medical College, Vellore, Humsafar Trust Mumbai, And The AIDS Malignancy Consortium (AMC). Correspondence to alexandra.hernandez@ucsf.edu.*

b	On average, in the past <u>30 days</u>, how often have you used a condom during <u>anal</u> intercourse with a <u>female</u>?	Never ☐ 1<br>1/month or < ☐ 2<br>1/week ☐ 3<br>3-4 /week ☐ 4<br>5-10 /week ☐ 5<br>11-20 /week ☐ 6<br>20+/week ☐ 7<br><br>Decline ☐ 99

Female Partner – Oral

**58.**	**Have you ever had <u>oral sex</u> with a <u>woman</u> until now (putting your tongue in or on your partner's vagina)?**	Yes ☐ 1 No ☐ 0<br>*If no skip to Q61*<br><br>Decline ☐ 99
a.	What is the number of female partners you have had <u>oral sex</u> with until now?	1-4 ☐ 1<br>5-9 ☐ 2<br>10-19 ☐ 3<br>20-39 ☐ 4<br>40+ ☐ 5<br><br>Decline ☐ 99
**59.**	**How many <u>Female</u> sexual partners have you performed oral sex on in the past <u>6 months</u>?**	0 ☐ 0<br>1 ☐ 1<br>2-4 ☐ 2<br>5-14 ☐ 3<br>15-29 ☐ 4<br>30-49 ☐ 5<br>50-99 ☐ 6<br>100+ ☐ 7<br><br>Decline ☐ 99
**60.**	**How many <u>Female</u> sexual partners have you performed oral sex on in the past <u>30 days</u>?**	0 ☐ 0<br>1 ☐ 1<br>2-4 ☐ 2<br>5-14 ☐ 3<br>15-29 ☐ 4<br>30-49 ☐ 5<br>50-99 ☐ 6<br>100+ ☐ 7<br><br>Decline ☐ 99

***HPV IN INDIA MEN (HIIM) Research Group** – A Collaboration among the University of California, San Francisco (UCSF), Christian Medical College, Vellore, Humsafar Trust Mumbai, And The AIDS Malignancy Consortium (AMC). Correspondence to alexandra.hernandez@ucsf.edu.*

K (continued): Sex work

Please read to the participant:

I am now going to ask you a few questions about sex work. Sex work is when money, goods or services are exchanged for any kind of sexual behavior.

**61.**	**Have you ever had sex with a <u>female</u> commercial sex worker?**	Yes ☐ 1 No ☐ 0<br>*If no skip to Q62*<br>Decline ☐ 99
	a. Have you had sex with a <u>female</u> commercial sex worker in the past <u>6 months?</u>	Yes ☐ 1 No ☐ 0<br>*If no skip to Q62*<br>Decline ☐ 99
	b. Have you had sex with a <u>female</u> commercial sex worker in the past 30 days?	Yes ☐ 1 No ☐ 0<br>Decline ☐ 99
**62.**	**Have you ever had sex with a <u>man</u> commercial sex worker?**	Yes ☐ 1 No ☐ 0<br>*If no skip to Q63*<br>Decline ☐ 99
	a. Have you had sex with a <u>man</u> commercial sex worker in the past <u>6 months?</u>	Yes ☐ 1 No ☐ 0<br>*If no skip to Q63*<br>Decline ☐ 99
	b. Have you had sex with a <u>man</u> commercial sex worker in the past <u>30 days</u>?	Yes ☐ 1 No ☐ 0<br>Decline ☐ 99
**63.**	**Have you ever had sex with a <u>Hijra/Arwani (or male-to-female transgender)</u>?**	Yes ☐ 1 No ☐ 0<br>*If no skip to Q64*<br>Decline ☐ 99
	a. Have you had sex with a <u>Hijra</u> in the past 6<u> months?</u>	Yes ☐ 1 No ☐ 0<br>*If no skip to Q64*<br>Decline ☐ 99
	b. Have you had sex with a <u>Hijra</u> in the past <u>30 days</u>?	Yes ☐ 1 No ☐ 0<br>Decline ☐ 99

HPV IN INDIA MEN (HIIM) Research Group *– A Collaboration among the University of California, San Francisco (UCSF), Christian Medical College, Vellore, Humsafar Trust Mumbai, And The AIDS Malignancy Consortium (AMC). Correspondence to alexandra.hernandez@ucsf.edu.*

**64.**	**Have you ever accepted money or goods to have sex with a man?**	Yes ☐ 1 No ☐ 0<br>*If no skip to conclusion*<br>Decline ☐ 99
a.	Did you accept money to have sex with a man in the past 6 months?	Yes ☐ 1 No ☐ 0<br>*If no skip to conclusion*<br>Decline ☐ 99
b.	Did you accept money to have receptive sex with a man in the past 6 months?	Yes ☐ 1 No ☐ 0<br>*If no skip to conclusion*<br>Decline ☐ 99
c.	Did you accept money to have sex with a man in the past 30 days?	Yes ☐ 1 No ☐ 0<br>*If no skip to conclusion*<br>Decline ☐ 99
d.	Did you accept money to have receptive sex in the past 30 days?	Yes ☐ 1 No ☐ 0<br>Decline ☐ 99

L: Conclusion

Please read to the participant:

This concludes the interview. Thank you very much for your help. If you have any questions at all about the study, please fell free to call [insert PM name and number from each site] or Dr. Joel Palefsky at UCSF 001-415-476-1574

**65.**	**Did the participant have any difficulty with the questionnaire?**	Yes ☐ 1 No ☐ 0
a.	If yes, please describe:____<br>____<br>____<br>____	
**66.**	**How confident are you of the validity of the answers?**	Not confident ☐ 1<br>Mildly confident ☐ 2<br>Fairly confident ☐ 3<br>Confident ☐ 4<br>Very confident ☐ 5
a.	If 1-3, please describe:____<br>____<br>____	

67. Interviewer Signature **Date**

____ ____________________
dd/mmm/yyyy

HPV IN INDIA MEN (HIIM) Research Group *– A Collaboration among the University of California, San Francisco (UCSF), Christian Medical College, Vellore, Humsafar Trust Mumbai, And The AIDS Malignancy Consortium (AMC). Correspondence to alexandra.hernandez@ucsf.edu.*
